# A decade of acquired aplastic anemia: insights from a Central South African Center

**DOI:** 10.11604/pamj.2025.50.86.44404

**Published:** 2025-03-28

**Authors:** Debi Mmasabata Seriti Moagi, Jacques Le Roux Malherbe, Claire Louise Barrett

**Affiliations:** 1 Department of Internal Medicine, School of Clinical Medicine, Faculty of Health Sciences, University of the Free State, Bloemfontein, South Africa,; 2 Research and Development Unit, School of Clinical Medicine, Faculty of Health Sciences, University of the Free State, Bloemfontein, South Africa

**Keywords:** Aplastic anaemia, haematological disorder, outcome, South Africa

## Abstract

**Introduction:**

aplastic anemia (AA) is a rare blood disorder with a considerable mortality rate, particularly if left untreated. Limited South African data on AA prompted this study to investigate the clinico-hematological features, management, and outcome of patients managed at our center.

**Methods:**

a ten-year retrospective observational study of all patients at the Universitas Academic Hospital with confirmed AA was conducted. Data on patient demographics, clinical and laboratory data, management, and outcomes were collected and analyzed.

**Results:**

twenty-seven patient files were included. Idiopathic AA was the most common (55.6%), while 12 patients (44.4%) had secondary causes. Most (70.4%) patients presented with severe AA. The majority (77.8%) received immunosuppressive therapy (IST) as first-line treatment, with most (80.9%) responding to treatment (complete, hematological and partial responses). Overall treatment response across available modalities was good (77.8%), with a few (22.2%) cases of refractory disease. Many patients were lost to follow-up (51.9%). Although access to hematopoietic stem cell transplantation (HSCT) in South Africa is limited, our center's treatment response rate to IST (80.9%) is comparable to international studies. The study found no association between HIV and AA, and no transfusion-related complications were observed.

**Conclusion:**

the study suggests that AA remains a disease of the young in South Africa. Most patients in this study presented with severe disease. The presence of paroxysmal nocturnal hemoglobinuria is in line with other studies. Addressing the high loss-to-follow-up rate is crucial for future studies.

## Introduction

Aplastic anemia (AA) is a rare blood disorder with a considerable mortality rate, particularly if left untreated [1,2]. This disorder is characterized by reduced or absent hematopoietic stem cell (HSC) precursors and hypocellular bone marrow [3,4]. The clinical manifestations are related to the cytopenias of the three cell lines, with anemia being the most prevalent, followed by bleeding and, less frequently, infection [5,6]. Aplastic anemia may be due to congenital or acquired causes. The acquired causes of AA are still poorly explained, and up to 80% of cases remain idiopathic. Certain drugs, environmental or occupational toxins, and viral infections are associated with acquired cases [7]. The exact pathophysiology of idiopathic AA is unclear, although in most cases, it is thought to be immune-mediated [8]. Activated cytotoxic T-cells are implicated in the immune destruction of HSCs and multipotential progenitor cells, which results in bone marrow failure, although the cause of T-cell activation is not well understood [8,9]. First described by Ehrlich in the late nineteenth century, AA has since been thoroughly researched and documented [10,11]. In South Africa, AA predominantly affects young people in their second and third decades of life. Still, contrary to international data, a clear second age peak in the fifth decade is not noted [12,13]. With the exception of two studies [12,13], data on adult AA patients in South Africa (SA) is scarce. It is crucial to investigate adult AA patients´ clinical presentation, treatment practices, and outcomes in a South African context to inform optimal management strategies within the SA healthcare system. This study aimed to provide epidemiological information regarding the clinical and hematological features, treatment, and outcomes of patients with AA at the Universitas Academic Hospital (UAH), a public sector, tertiary hospital affiliated with the University of Free State (UFS), in Bloemfontein, Free State, SA.

## Methods

**Study design:** a single-center, retrospective observational and analytical study was performed.

**Setting:** the study was performed at the Clinical Hematology Unit, Department of Internal Medicine, Universitas Academic Hospital (UAH), Bloemfontein, South Africa. This tertiary center provides clinical hematology services to patients in the Free State, Northern Cape, parts of the Eastern Cape, and the neighboring country of Lesotho.

**Participants:** all records of patients (12 years and older) seen at the Clinical Hematology Unit at UAH with a confirmed diagnosis of AA (of any severity) according to the modified Camitta criteria [14] between 1^st^ January 2012 and 31^st^ December 2021 were included in the study. Records of patients younger than 12 years, patients who did not fulfil the diagnostic criteria for AA, and patients with genetic conditions leading to congenital AA (e.g., Fanconi anemia, Diamond-Blackfan anemia, dyskeratosis congenita, Shwachman-Diamond syndrome) were excluded. All patient records archived at the Clinical Hematology Outpatient Clinic and the Clinical Hematology ward admission register were reviewed to identify patients with a diagnosis of AA. Pharmacy records of anti-thymocyte globulin (ATG) (first-line IST for AA at our unit) were also used to trace patients treated for AA.

**Outcomes:** hematological response was defined a: i) independence from transfusions and ii) improvement of peripheral blood counts to at least two of the following: neutrophils > 0.5×10^9^/L, platelets > 20x10^9^/L or reticulocyte count > 20×10^9^/L. Complete response was defined as: i) independence from transfusions and ii) improvement of peripheral blood hemoglobin > 10 g/dL together with a platelet count > 50×10^9^/L. A partial response was defined as a clear improvement in the blood counts and/or transfusion requirements that did not meet the criteria for a complete response. Refractory disease was defined as a lack of significant improvement in cytopenias and/or transfusion requirements [15].

**Statistical analysis:** the study data was collected and managed using REDCap. Data analysis was performed by a biostatistician at the UFS using SAS, version 9.4 (SAS Institute Inc.; Cary, NC, United States [US]). Categorical data were summarized using frequencies and percentages. Continuous variables were described using the mean and standard deviation or median and interquartile range when variables were not normally distributed. Groups were compared using the Student´s t-test (parametric or normally distributed data) and Kruskal-Wallis test (non-parametric or data that was not normally distributed) for continuous variables and the chi-square test for proportions. A p-value of < 0.05 was considered statistically significant.

**Ethical considerations:** ethical clearance for the study was obtained from the Human Research Ethics Committee of the University of Free State, Bloemfontein (UFS-HSD2022/0693/2607). Approval to conduct the study was also obtained from the Head of Internal Medicine at UAH and the Free State Provincial Health Department. No identifying patient data were collected. All data collected were encrypted and stored on a password-protected device.

## Results

Seventy-two records were identified and screened for eligibility in the study. Eighteen records could not be located. A further 27 records were excluded due to incorrect diagnosis, congenital AA, patients younger than 12 years at diagnosis, or records falling outside the study period. A total of 27 records were included in the final analysis.

**Demographics:** the sex distribution in the study was almost equal, although a slight female predominance (n= 14/27, 51.9%) was noted. The median age was 23 years (range: 13-60 years). The black African population was the largest representation (n= 21/27, 77.8%), while 3 (11%) white and 3 (11%) mixed-race patients completed the sample.

**Clinical presentation:** most patients presented with fatigue (n= 16/27, 59.3%), followed by complaints of bleeding (n= 11/27, 40.7%), with vaginal bleeding (n= 7/11, 63.6%) being the most common site of bleeding. Fever (n= 2/27, 7.4%) and night sweats (n= 1/27, 3.7%) were less commonly reported.

**Baseline and last-visit laboratory parameters:** Table 1 presents the relevant full blood count (FBC) findings at baseline and at the last clinic visit. The mean hemoglobin at diagnosis was 5.5 g/dL (range: 2.3 - 9.9 g/dL). Nearly half presented with hemoglobin < 5.0 g/dL (n= 11/27, 40.7%) at diagnosis. The mean hemoglobin at the last visit was higher (9.5 g/dL). A similar trend was observed in the platelet counts, with the mean increasing at baseline from 10×10^9^/L to 47×10^9^/L at the last visit. The change in mean absolute white cell count (WCC) at baseline (2.31×10^9^/L) compared to the last visit value was not as marked (median WCC= 2.73×10^9^/L). Neutrophil counts improved markedly from a median of 0.57 x10^9^/L at baseline to almost three times the value (1.49×10^9^/L) at the last visit.

**Table 1:** baseline and last visit full blood count

	Baseline		Last clinic visit	
	n	mean (range)	n	mean (range)
Hemoglobin (g/dL)	27	5.5 (2.3-9.9)	27	9.5 (3.1-15.6)
Platelets (x 10^9^/L)	27	10* (1-139)	27	47* (5-273)
Absolute white cell count (x 10^9^/L)	27	2.31 (1.32-4.27)	27	2.73* (0.54- 9.73)
Neutrophil count (x 10^9^/L)	27	0.57* (0- 4.81)	27	1.49* (0.03-9.0)
Lymphocyte count (x 10^9^/L)	27	1.33 (0.38 – 3.07)	27	1.09* (0.19 – 3.71)
Absolute reticulocyte count (x 10^12^/L)	15	0.022* (0.002- 0.124)	0	

* Median; FBC: full blood count

**Etiology/associations:** Table 2 summarizes the etiological associations identified in our study population. Most cases of AA were idiopathic, with no identifiable cause/association in over half of the patient population (n= 15/27, 55.6%). A few patients (n= 2/27, 7.4%) had an underlying occupational or environmental exposure. Two patients (7.4%) had a positive anti-nuclear antibody (ANA). However, not enough data was available to establish the European League Against Rheumatism and the American College of Rheumatology (EULAR/ACR) criteria [16] for diagnosing systemic lupus erythematosus (SLE). One patient (3.7%) tested positive for parvovirus-B19, and none had active hepatitis. A few patients (n= 2/27, 7.4%) were persons living with HIV/AIDS (PLWHA). Their CD4 counts were 54 cell/μL and 696 cell/μL, respectively, with both patients having a suppressed HIV viral load (< 50 copies/mL). Both patients were reported to be taking combined antiretroviral therapy (cART). One patient was taking lamivudine (3TC) as part of the cART, with zidovudine (AZT) not forming part of either of the patients´ treatment regimens. Concomitant use of drugs associated with AA was found in a few of the patient population (n= 5/27, 18.5%), with some patients taking more than one concomitant drug. These drugs include anti-hypertensives, anti-depressants, anti-thyroid, anti-epileptics, antibiotics, illicit drugs and traditional medications. None of the patients had a family history of hematological disorders. Of the 14 female patients, pregnancy was excluded through a qualitative serology test forβ-HCG in 10 patients (71.4%), while no results were found for the remaining patients (n= 4, 28.6%).

**Table 2:** etiological associations identified

		n (%)
**Associations identified (n=27)**		
	HIV positive	2 (7.4)
	ANA positive	2 (7.4)
	Environmental exposure	2 (7.4)
	Possible drug cause	5 (18.5)
	Parvovirus B19	1 (3.7)
	Active hepatitis	0 (0)
	Pregnancy	0 (0)
**No association identified (n=27)**		
	No cause/association identified	15 (55.6)

HIV: human immunodeficiency virus; ANA: anti-nuclear antibody

**Disease severity:** the modified Camitta criteria were used for this study to assess disease severity [14]. More cases of severe disease (n= 19, 70.4%; males: 10, females: 9) were observed compared to non-severe (n= 7, 25.9%; males: 3, females: 4). An interesting finding is that of no cases of very severe aplastic anemia (VSAA), which may have been influenced by the large number of missing records. This is not in line with the studies performed in Gauteng Province [12,13].

**Management of patients with a confirmed diagnosis of aplastic anemia:** the standard IST at our center is a combination of equine anti-thyomocyte globulin (ATG) + cyclosporin A (CsA) with the addition of steroids to prevent serum sickness. Table 3 presents the breakdown of treatment received by the patients as first-line therapy. Most patients (n= 21/27, 77.8%) received IST as first-line therapy, whereas just five patients (18.5%) received the androgen danazol (Table 3). Non-severe aplastic anemia (NSAA) was present in 80.0% (n= 4/5) of patients who received danazol. The fifth patient had severe aplastic anemia (SAA), but it was not possible to determine from the documentation why they were given danazol. In addition, NSAA was present in the patient for observation. In three patients (11.1%), granulocyte colony-stimulating factors (G-CSF) were given as supportive therapy. It is significant to note that two patients were transferred to Cape Town for hematopoietic stem cell transplantation (HSCT); however, they were excluded from the study due to incomplete records and a missing file.

**Table 3:** first-line therapy

	N (%)
Immunosuppressive therapy (ATG + CsA)	21 (77.8)
Other (Danazol)	5 (18.5)
Bone marrow transplant	0 (0)
Observation	1 (3.7)

ATG: anti-thymocyte globulin; CsA: cyclosporin A

**Outcomes:** treatment response was categorized as complete, hematological, partial response, and refractory disease. Table 4 below shows an overview of the overall treatment response. The time to relapse from the best response was six months in one patient and 16 months in the second patient. Just over half of the patients in our study were lost to follow-up (n= 14/27, 51.9%). Of the patients lost to follow-up, most had a documented partial response to therapy (n= 8/14, 57.1%), while some (n= 4/14, 28.6%) had a complete response to therapy. Only a few of the patients that were lost to follow-up (n= 2/14, 14.3%) had not responded to treatment at their last known visit. Almost half the patient population (n= 12/27, 44.4%) was alive at the end of the study period, and approximately two-thirds of these patients had SAA at diagnosis. One patient with SAA died. The cause of death in this patient was neutropenic sepsis, which occurred three months after the diagnosis of AA.

**Table 4:** treatment response and outcome

		N (%)
**Treatment response (n=27)**	Complete response	12 (44.4)
	Hematological response	2 (7.4)
	Partial response	7 (25.9)
	Refractory disease	6 (22.2)
**Disease relapse (n=27)**	Yes	2 (7.4)
	No	22 (81.5)
	Unknown	3(11.1)
**Outcome**	Alive	12(44.4)
	Dead	1 (3.7)
	Lost to follow up	14 (51.9)

**Transfusion requirements:** approximately two-thirds of the patient population received a transfusion of red cell concentrate and platelet products (n= 18/27, 66.7% and n= 17/27, 63.0% respectively) at the Clinical Haematology Unit. Sixty-seven units of leucodepleted (non-irradiated) red cell concentrate and 29 units of leucodepleted irradiated red cell concentrate were transfused to 11 patients. A mean of five units of leucodepleted (non-irradiated) red cell concentrates and a mean of five units of platelet concentrate were transfused per patient. Of the 18 patients who received red cell concentrate transfusions, a small number (n= 4/18, 22.2%) received exclusively irradiated red cell concentrate, and a third (n=6/18, 33.3%) received only leucodepleted, non-irradiated red cell concentrates. The remainder received both irradiated and non-irradiated red cell concentrate. Data regarding transfusions that patients received at their base hospitals were not collected. However, the majority (n= 20/27, 74.1%) received transfusions at their base hospitals. The details of which blood products were administered to these patients are unknown. No patients had suspected transfusion-associated graft versus host disease (TA-GVHD) or CMV reactivation. A cautionary note, however, is that a large proportion of our patient population was lost to follow-up, and formal investigation for TA-GVHD was not performed in any patient.

**Transfusion-related iron overload:** for the study, iron overload refers to a serum ferritin level of 200-300 μg/L or more and transferrin saturation of 45% or more. Just over two-thirds (n= 12/18, 66.7%) of the patients who received red cell products met the study criteria for iron overload according to serum ferritin and transferrin saturation levels; the remainder either did not meet the criteria for transfusion-related iron overload or did not have ferritin and/or transferrin saturation testing performed (3/18, 16.5% each). However, no patients had documented clinical features of iron overload, and no iron chelation therapy was used.

**Cytogenetic abnormalities:** most patients (n= 20/27, 74.1%) were tested for Fanconi anemia (FA) at diagnosis, of which 19 (95.0%) tested negative, and the results for one patient could not be traced. A clonal disorder was present in over a third (n= 10/27, 37.0%) of the patients. Paroxysmal nocturnal hemoglobinuria was the most common clonal disorder in this group of patients (n = 9/10, 90.0%), followed by secondary myelodysplastic syndrome (MDS) in one patient (n= 1/10, 10.0%). A few (n= 5/27, 18.5%) patients underwent cytogenetic testing. Telomerase reverse transcriptase (TERT) and Telomerase RNA Component (TERC) mutations were tested for in two patients, both of which were negative for these mutations (n= 2/2, 100%). No abnormal karyotypes were detected in the tested patients (n = 2/2, 100%). No evidence of monosomy 7 was found in the patient for whom testing was done (n= 1/1, 100%).

**Paroxysmal nocturnal hemoglobinuria (PNH) clones:** testing for PNH clones was performed on most patients (n= 22/27, 81.5%) at diagnosis, and PNH clones were detected in a few (n= 4/22, 18.2%) of those tested. Repeat testing for PNH was performed at various time intervals during follow-up. Repeat testing for PNH revealed a positive result in 6 patients who previously tested negative for PNH. Overall, PNH clones were present in nearly half the tested patient population (n= 10/22, 45.5%).

**Role of HIV status, disease severity and paroxysmal nocturnal hemoglobinuria clones on treatment response**

**HIV and treatment response:** the relationship between HIV, disease severity at diagnosis and treatment response was assessed. The results of this correlational analysis were limited due to the small number of HIV-positive patients (n= 2/27, 7.4%). The analysis showed that 1 of the 2 (50%) HIV-positive patients and 18 of 23 (78.3%) HIV-negative patients presented with severe AA. Similarly, 1 of the 2 HIV-positive patients (50%) and 4 of 23 (17.4%) HIV-negative patients presented with non-severe AA. Additionally, none of the PLWHA showed a complete response to treatment, while a complete and hematological response rate of over 50% was noted in the HIV-negative group (p= 0.026). The clinical significance of these results is dubious given the small sample size of PLWHA.

**Disease severity and treatment response:** patients with SAA had a higher rate of refractory disease (n= 6/19, 31.6%), while almost half of those with NSAA showed complete response (n= 3/7, 42.9%) and no refractory disease (0.0%) (p= 0.0039). The relation between treatment response and disease severity was statistically significant.

**Paroxysmal nocturnal haemoglobinuria and treatment response:** fewer cases of the refractory disease were observed in patients with a PNH clone at baseline (n= 1/5, 20.0%) compared to those who did not have a PNH clone at baseline (n= 4/5, 80.0%). However, this was not statistically significant (p= 0.09).

## Discussion

This retrospective study comprised 27 patient files from a resource-limited academic hospital in the Free State, South Africa. While this number is small compared to other South African centers (2.7 compared to 5.8 - 6.4 new patients per year in Gauteng), the Free State serves a much smaller population than the other South African centers cited [12,13]. Correcting for the size of the provinces (a population of just over 2.8 million in the Free State versus a 15.1 million population in Gauteng province), about 0.96 cases per million cases were seen in the Free State public sector, and between 0.38 - 0.42 cases per million in Gauteng [17,18]. There seems to be a higher prevalence of AA in our study compared to the two Gauteng studies. The prevalence reported in our study and the two other South African AA studies is still much lower than reported in Europe and North America [19]. We believe that the prevalence of AA may be higher than indicated in this study, as there were many missing records. Furthermore, patients from the Free State must travel large distances between referral centers and our hospital, resulting in patient transport challenges and poor access to primary health care [20]. Aplastic anemia appears to be a disease of the young in South Africa, with no clear second age peak noted in our study population. Aplastic anemia is typically described as having a bimodal age distribution in international literature, with the first age peak usually occurring in the second and third decades of life and the second age peak occurring above 60 years of age [21]. This finding of no clear second age peak is similar to other South African studies [12,13]. It is not well understood why this second age peak is not observed in our setting. One plausible explanation is that the life expectancy in South Africa is comparatively lower than that of high-income countries (HICs) [22]. Much of our study population was black Africans, which is in keeping with the population of the region served. Due to the small sample size and missing records, these observations should be interpreted cautiously. The equal gender distribution noted in this study aligns with most international studies. However, the male predominance, particularly in patients younger than 40 years of age, reported elsewhere, was not noted in our study [19,23].

Most patients had severe disease (SAA) at presentation, with more males presenting with severe disease than females. Our findings are in line with other research, which shows that the initial age peak in AA is primarily made up of young males with more severe disease compared to females, while the second age peak (> 60 years) reported elsewhere is primarily made up of females with non-severe disease [24,25]. The bimodal age distribution can be explained by variations in host susceptibility, exposure to risk factors, and bone marrow reserves in the different age groups [26]. South Africa has a high HIV prevalence, estimated at 13.7% of the total population [27]. Despite the noted increase in prevalence in PLWHA in SA from an estimated 3.8 million persons in 2002 to 8.2 million persons by 2021 [27], only two patients in our study population were HIV positive, both of whom had a suppressed HIV viral load. The prevalence of HIV in our study population is, therefore, much lower than the national prevalence. Our sample size is small, and this finding might not be representative of the national population. In South Africa, blood products are not routinely tested for cytomegalovirus (CMV) [28]. While the British Committee for Standards in Hematology (BCSH) guidelines recommend using CMV-negative blood products for patients with AA, this cannot be practiced locally, so no patients in this study received CMV-negative blood products [14]. Interestingly, more patients received leucodepleted (non-irradiated) red cell concentrate compared to irradiated red cell concentrates in this study. There is a paucity of information on using irradiated blood products in AA, and practices guiding their usage vary [29]. In AA, irradiated blood products are recommended to lower the risk of alloimmunization and to prevent TA-GVHD in patients receiving ATG [7,29].

In South Africa, irradiated red cell products need to be ordered on a named patient basis and are not available for immediate use in all blood banks. There are a limited number of irradiation sites nationally, and these are situated in larger urban centers. This may result in delays in issuing irradiated red cell products to smaller centers. Irradiated platelet products are less of a challenge, as pre-irradiation transfusion cross-matching is not required when issuing platelet products. All single-donor apheresis platelet concentrates in SA are routinely leucodepleted. No cases of CMV reactivation or TA-GVHD were reported in this study. However, this should be interpreted with caution due to the high number of patients who were lost to follow-up. Due to our institution's limited access to HSCT, most of our patients were treated with first-line IST, comprising ATG (equine) and CsA. The response to treatment in our study was comparable with international studies, with an IST response rate (complete and partial) of almost 80% [19,23]. This finding is consistent with earlier research, which indicated that younger patients respond better to IST, which is especially important given that our study cohort was relatively young [24]. The presence of PNH clones has been reported to have a favorable outcome to IST [30,31]. Although not statistically significant, fewer cases of refractory disease were seen in PNH-positive patients, likely related to the small sample size. Furthermore, more than half of our PNH-positive population was picked up at the second test, which may support performing repeat PNH testing at set time intervals in the monitoring of patients, as seen in some international literature [32]. Only one patient in our study died. A significant number of patient records were not traceable, and many patients were lost to follow-up. Reasons for this could include patients continuing care at their referring hospitals, dying at home, poor socioeconomic status, distances to specialist centers, and even the effects of the Coronavirus disease 2019 (COVID-19) pandemic. The COVID-19 pandemic has significantly impacted healthcare globally [33]. Patients with rare diseases have always suffered from a lack of resources; the pandemic heightened this. Face-to-face consultations decreased during the lockdown periods. Furthermore, the notion that patients with rare diseases were at higher risk of contracting and succumbing to COVID-19 complications led to more patients staying at home [33,34]. These factors make calculating an accurate mortality rate impossible.

**Limitations:** this study is limited due to its retrospective nature, missing records and incomplete data, and the large number of patients that were lost to follow-up.

**Generalizability:** the small sample size did not allow statistical significance generation for several variables. It was also impossible to assess and compare disease severity and treatment responses between PLWHA and non-infected individuals, given the small number of PLWHA in our study. While two patients were referred for HSCT, their records could not be included in the study. Therefore, we could not assess the response to HSCT and compare this treatment modality with other existing modalities at the institution and with other studies. More importantly, the determination of true mortality was limited by the many patients lost to follow-up.

## Conclusion

In the South African setting, AA remains a disease of the young, most of whom present with severe disease. Furthermore, contrary to internationally published studies, no clear second age peak was observed. Access to HSCT in SA is still quite limited, although our treatment response rate to IST is comparable with international studies, with rates of 80.9%. Additionally, repeat testing for PNH at set time intervals may be incorporated into patient care, as more than half of the cases can be missed at initial diagnosis. There were no reported cases of CMV reactivation or TA-GVHD. The mortality rate in our study could not be determined, largely due to the many patients lost to follow-up. This may be an area of further research.

### What is known about this topic

- Aplastic anemia is a rare blood disorder with a considerable mortality rate, particularly if left untreated;
- In South Africa, aplastic anemia predominantly affects young people in their second and third decades of life;
- Data on adult aplastic anemia patients in South Africa is scarce.

### What this study adds

- Access to hematopoietic stem cell transplantation in South Africa is still quite limited, although our treatment response rate to immunosuppressive therapy is comparable with international studies;
- Repeat testing for paroxysmal nocturnal hemoglobinuria at set time intervals may be incorporated into patient care, as more than half of the cases can be missed at initial diagnosis.
